# Exploring the Neighborhood of *q*-Exponentials

**DOI:** 10.3390/e22121402

**Published:** 2020-12-11

**Authors:** Henrique Santos Lima, Constantino Tsallis

**Affiliations:** 1 Centro Brasileiro de Pesquisas Físicas, Rua Xavier Sigaud 150, Rio de Janeiro, RJ 22290-180, Brazil; tsallis@cbpf.br; 2 National Institute of Science and Technology of Complex Systems, Rua Xavier Sigaud 150, Rio de Janeiro, RJ 22290-180, Brazil; 3 Santa Fe Institute, 1399 Hyde Park Road, Santa Fe, NM 87501, USA; 4 Complexity Science Hub Vienna, Josefstädter Strasse 39, 1080 Vienna, Austria

**Keywords:** q-exponentials, nonextensive statistical mechanics, nonadditive entropies, complex systems

## Abstract

The *q*-exponential form $e_{q}^{x}\equiv {\left[1+\left(1-q\right)x\right]}^{1/(1-q)}\;\;\left(e_{1}^{x}=e^{x}\right)$ is obtained by optimizing the nonadditive entropy $S_{q}\equiv k\frac{1-\sum_{i}p_{i}^{q}}{q-1}$ (with $S_{1}=S_{BG}\equiv -k\sum_{i}p_{i}\ln p_{i}$, where BG stands for Boltzmann–Gibbs) under simple constraints, and emerges in wide classes of natural, artificial and social complex systems. However, in experiments, observations and numerical calculations, it rarely appears in its pure mathematical form. It appears instead exhibiting crossovers to, or mixed with, other similar forms. We first discuss departures from *q*-exponentials within crossover statistics, or by linearly combining them, or by linearly combining the corresponding *q*-entropies. Then, we discuss departures originated by double-index nonadditive entropies containing $S_{q}$ as particular case.

## 1. Introduction

Nonadditive entropies have been used as a basis to explain a diversity of phenomena, from astrophysics to the oscillatory behavior of El Niño [1,2,3], from DNA to financial markets [4,5] from high-energy physics of collisions to granular matter and cold atoms [6,7,8], among many others. It turns out that wide classes of complex systems can be satisfactorily handled within a generalization of Boltzmann–Gibbs (BG) statistical mechanics based on the nonadditive entropy

(1) $$S_{q}\equiv k\frac{1-\sum_{i=1}^{W}p_{i}^{q}}{q-1}=k\sum_{i=1}^{W}p_{i}\ln_{q}\frac{1}{p_{i}}\;\;\;\;\left(q\in R;\;S_{1}=S_{BG}\equiv -k\sum_{i=1}^{W}p_{i}\ln p_{i};\;\sum_{i=1}^{W}p_{i}=1\right)\;,$$

where *W* is the total number of microstates and *k* is a conventional positive constant (usually $k=k_{B}$ in physics, and k=1 in computational sciences), the *q*-logarithmic function being defined as $\ln_{q}z\equiv \frac{z^{1-q}-1}{1-q}$ ($\ln_{1}z=\ln z$). This theory is currently referred to as nonextensive statistical mechanics, or *q*-statistics for short [9,10,11]. The optimization of $S_{q}$ with simple constraints yields

(2) $$p_{i}=\frac{e_{q}^{-\beta_{q}E_{i}}}{\sum_{j=1}^{W}e_{q}^{-\beta_{q}E_{j}}}\;,$$

where $\left\{E_{i}\right\}$ are the energy eigenvalues, and the *q*-exponential function (inverse of the *q*-logarithmic function) is defined as follows:

(3) $$e_{q}^{x}\equiv {\left[1+\left(1-q\right)x\right]}_{+}^{\frac{1}{1-q}}\;\;\;\left(q\in R;\;e_{1}^{x}=e^{x}\right)\;,$$

where ${\left[z\right]}_{+}=z$ if z>0 and zero if z≤0; notice that this definition implies that, for q<1, there is a cutoff at $x_{cutoff}=-1/\left(1-q\right)<0$ [9]. In the limit q→1, Equation (2) recovers the celebrated BG weight.

The aim of the present article is to discuss in detail some departures from a pure *q*-exponential function which frequently emerge in real situations. Such variations are used in the statistics of nucleotides in full genomes [4], the re-association of folded proteins [12], standard map for intermediate values of the control parameter [13], to mention but a few. We focus on crossover statistics (Section 2), linear combinations of *q*-exponential functions (Section 3), linear combinations of *q*-entropies (Section 4), and some two-indices entropies, namely $S_{q,\delta }$[14], $S_{q,q^{\prime }}^{BR}$[15] and $S_{q,q^{\prime }}$ [16] (Section 5).

## 2. Multiple Crossover Statistics

Crossover statistics is often useful whenever the phenomenon which is focused on exhibits a *q*-exponential behavior within a range of the relevant variables, and then makes a crossover to another *q*-exponential function with a different index *q*. Although rare, it can, in principle, happen that several crossovers successively occur one after the other. We will refer to it as *multiple crossover statistics*.

Illustrations of such crossovers can be found in [12,17,18,19,20,21].

Let us consider the following ordinary differential equation:

(4) $$\frac{dy}{dx}=-ay^{q}\;\;\;\left(y\left(0\right)=1;\;a\in R\right)\;.$$

Its solution is given by

(5) $$y\left(x\right)=e_{q}^{-a\;x}\;.$$

Multiple crossovers emerge from the following nonlinear ordinary differential equation:

(6) $$\frac{dy}{dx}=-\sum_{k=1}^{M}a_{k}\;y^{q_{k}}\;\;\left(q_{1}<q_{2}<\cdots <q_{M}\right)\;,$$

with y(0)=1, and $0\leq a_{1}\leq a_{2}\leq \cdots \leq a_{M}$, where the right-hand term is constituted by a linear combination of nonlinear terms. Consequently

(7) $$x=\int_{y}^{1}\frac{dz}{\sum_{k=1}^{M}a_{k}\;z^{q_{k}}}\;.$$

**Figure 1:**
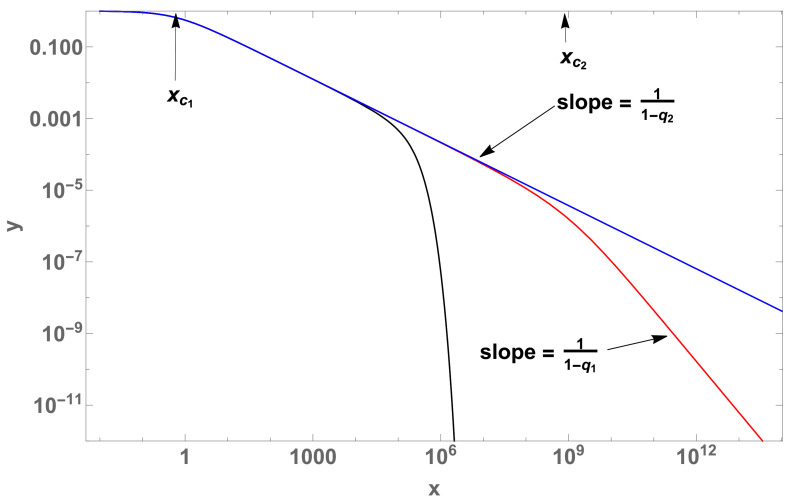
y(x) (log-log plot). For the case M=1 with (q,a)=(2.7,1) (blue curve) and, for the case M=2, the crossover between two curves, namely with $q_{1}=1$ (black curve) and $q_{1}=1.7$ (red curve) respectively, both with $\left(q_{2},\lambda_{q_{2}},\mu_{q_{1}}\right)=\left(2.7,1,1\times 10^{-5}\right)$. For the red curve, we have the crossover characteristic values $\left(x_{c_{1}},x_{c_{2}}\right)=\left(0.588,8.407\times 10^{8}\right)$, which indicate the passage from one regime to another.

We know that Equation (7) has analytical solutions for M=1 and M=2 (Figure 1). For M>2, we need to solve this equation numerically.

Particularly for crossover between two curves (M=2) with $q_{1}$ and $q_{2}$, we have:

(8) $$\frac{dy}{dx}=-a_{1}y^{q_{1}}-a_{2}y^{q_{2}}=-\mu_{q_{1}}y^{q_{1}}-\left(\lambda_{q_{2}}-\mu_{q_{1}}\right)y^{q_{2}}\;\;\;\left(y\left(0\right)=1\right)\;,$$

where we have identified $\left(a_{1},a_{2}\right)\equiv \left(\mu_{q_{1}},\lambda_{q_{2}}-\mu_{q_{1}}\right)$ in order to facilitate the connection with the notation used in [12]. Let us incidentally mention that this equation enabled the study of the anomalous behavior of folded proteins.

To solve Equation (8), we use Equation (7), which yields

(9) $$\begin{array}{c} \begin{array}{cc} x & =\frac{1}{\mu_{q_{1}}}\left\{\frac{y^{1-q_{1}}-1}{q_{1}-1}-\frac{\left(\frac{\lambda_{q_{2}}}{\mu_{q_{1}}}\right)-1}{1+q_{2}-2q_{1}}\right.\\ \; & \;\times \left[H\left(1;q_{2}-2q_{1},q_{2}-q_{1},\left(\frac{\lambda_{q_{2}}}{\mu_{q_{1}}}\right)-1\right)\right.\\ \; & \left.-H\left.\left(y;q_{2}-2q_{1},q_{2}-q_{1},\left(\frac{\lambda_{q_{2}}}{\mu_{q_{1}}}\right)-1\right)\right]\right\} \end{array} \end{array}$$

with

(10) $$H\left(y;a,b,c\right)=y^{1+a}{}_{2}F_{1}\left(\frac{1+a}{b},1;\frac{1+a+b}{c};-y^{b}c\right)\;,$$

where ${}_{2}F_{1}$ is a hypergeometric function.

For the particular case $q_{1}=1$, we obtain

(11) $$y=\frac{1}{{\left[1-\frac{\lambda_{q_{2}}}{\mu_{1}}+\frac{\lambda_{q_{2}}}{\mu_{1}}\;e^{\left(q_{2}-1\right)\;\mu_{1}x}\right]}^{\frac{1}{q_{2}-1}}}\;.$$

It is certainly worth mentioning that its $q_{2}=2$ instance yields $y={\left[1-\frac{\lambda_{2}}{\mu_{1}}+\frac{\lambda_{2}}{\mu_{1}}\;e^{\mu_{1}x}\right]}^{-1}$, whose $\lambda_{2}/\mu_{1}>>1$ asymptotic behavior becomes $y\propto 1/[e^{\mu_{1}\;x}-1]$. It is precisely through this ordinary-differential path that Planck found, in his historical 19 October 1900 paper, the thermostatistical factor which eventually led to his celebrated law for the black-body radiation with the ultimate identification $\mu_{1}x\to h\nu /k_{B}T$ [22,23].

For the case M=3, we have

(12) $$\frac{dy}{dx}=-a_{1}y^{q_{1}}-a_{3}y^{q_{2}}-a_{3}y^{q_{3}}$$

whose analytical solution is intractable. Therefore, we use numerical methods to solve it. In contrast, the characteristic values $\left(x_{c_{1}},x_{c_{2}},x_{c_{3}}\right)$ where changes of behavior of the curve occur are analytically accessible. Those values are obtained through the following considerations. For the characteristic value $x_{c_{1}}$, we have

(13) $$y\left(x_{c_{1}}\right)∼{\left[\left(q_{3}-1\right)a_{3}x_{c_{1}}\right]}^{-\frac{1}{q_{3}-1}}∼1\;.$$

Consequently

(14) $$x_{c_{1}}=\frac{1}{\left[\left(q_{3}-1\right)a_{3}\right]}\;.$$

For $x_{c_{2}}$ we have

(15) $$y\left(x_{c_{2}}\right)∼{\left[\left(q_{2}-1\right)a_{2}x_{c_{2}}\right]}^{-\frac{1}{q_{2}-1}}∼{\left[\left(q_{3}-1\right)a_{3}x_{c_{2}}\right]}^{-\frac{1}{q_{3}-1}}\;,$$

hence

(16) $$x_{c_{2}}=\frac{{\left[\left(q_{3}-1\right)a_{3}\right]}^{\frac{q_{2}-1}{q_{3}-q_{2}}}}{{\left[\left(q_{2}-1\right)a_{2}\right]}^{\frac{q_{3}-1}{q_{3}-q_{2}}}}\;.$$

Similarly, we have

(17) $$x_{c_{3}}=\frac{{\left[\left(q_{2}-1\right)a_{2}\right]}^{\frac{q_{1}-1}{q_{2}-q_{1}}}}{{\left[\left(q_{1}-1\right)a_{1}\right]}^{\frac{q_{2}-1}{q_{2}-q_{1}}}}\;.$$

**Figure 2:**
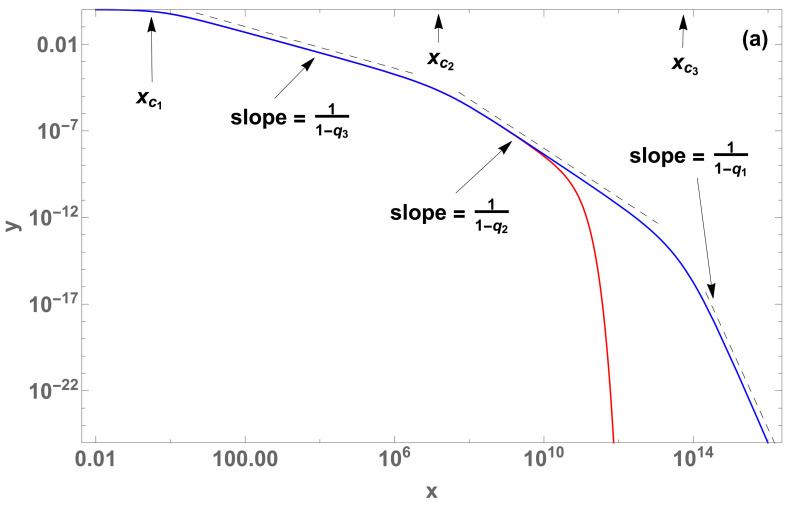

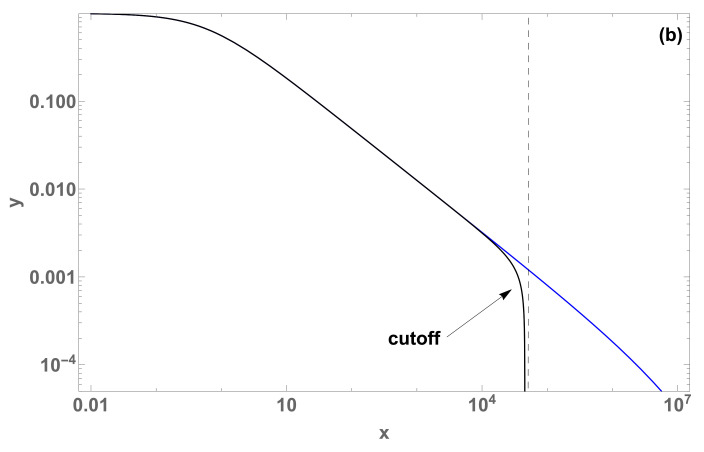
Crossovers in y(x) for M=3 (log-log plots) (**a**) between two curves with $\left(q_{1},q_{2}\right)=\left(1,1.7\right)$ (red curve), $\left(q_{1},q_{2}\right)=\left(1.2,1.7\right)$ (blue curve) respectively, both with $\left(q_{3},a_{1},a_{2},a_{3}\right)=(2.7,$$5\times 10^{-11}$,$1\times 10^{-4}$,1), and (**b**) a change was done on the blue curve, with $q_{1}=-1$ (black curve); the cutoff occurs at $x_{cutoff}\approx 4.48\times 10^{4}$.

Therefore, for the M=3 particular case whose parameter values are $a_{1}=5\times 10^{-11},\;a_{2}=1\times 10^{-4}$ and $a_{3}=1$, with $q_{1}=1.2,\;q_{2}=1.7$ and $q_{3}=2.7$, we have $x_{c_{1}}\approx 0.59$, $x_{c_{2}}\approx 1.68\times 10^{7}$ and $x_{c_{3}}\approx 5.47\times 10^{13}$, as shown in Figure 2a,b. It is similarly possible to study multiple crossovers for the case M>3.

## 3. Linear Combination of Normalized *q*-Exponentials

For a linear combination of normalized *q*-exponentials, we consider a probability distribution function P=P(x), $x\in X\subset R^{+}$ such that:

(18) $$P\left(x\right)=\sum_{k=1}^{M}b_{k}\;p_{k}\left(x\right)=\sum_{k=1}^{M}b_{k}\;\frac{e_{q_{k}}^{-\beta_{q_{k}}\;x}}{Z_{q_{k}}}\;\;\left(q_{1}\leq q_{2}\leq \cdots \leq q_{M}<2;\;\beta_{q_{k}}>0\;,\forall \;k\right)\;,$$

with $\sum_{k=1}^{M}b_{k}=1\;\;\left(b_{k}\geq 0\right)$, $\left\{Z_{q_{k}}\right\}$ being normalization factors (the upper limit q<2 emerges in order to $\left\{Z_{q_{k}}\right\}$ being finite). Those quantities are determined by imposing, for all k∈{1,...,M},

(19) $$\begin{array}{ccc} \int_{0}^{\infty }dx\;p_{k}\left(x\right) & = & 1\;\;if\;\;q_{k}\geq 1\;, \end{array}$$

(20) $$\begin{array}{ccc} \int_{0}^{\frac{1}{\beta_{q_{k}}\left(1-q_{k}\right)}}dx\;p_{k}\left(x\right) & = & 1\;\;if\;\;q_{k}<1\;. \end{array}$$

It follows

(21) $$Z_{q_{k}}=\frac{1}{\beta_{q_{k}}\left(2-q_{k}\right)}\;,\;\;\forall \;q_{k}<2\;.$$

**Figure 3:**
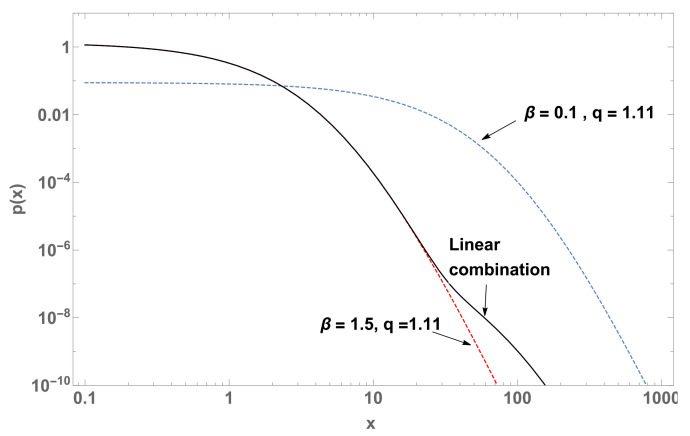
p(x) (log-log plot) of three curves (case M=2) with parameters q=1.11 and β=0.1 (blue dashed curve), β=1.5 (red dashed curve), and their linear combination (black curve) with $b_{1}=1\times 10^{-5}$ and $b_{2}=1-b_{1}$.

**Figure 4:**
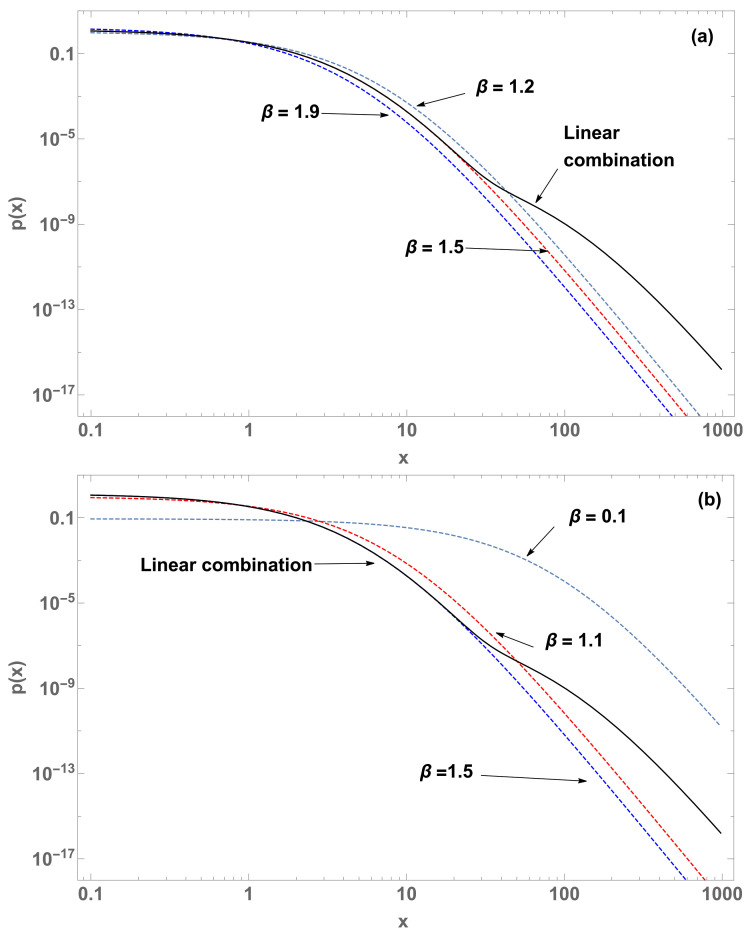
p(x) (log-log plots) of four curves with parameters q=1.11, β=1.9 (blue dashed curve), β=1.5 (red dashed curve), β=1.2 (gray dashed curve), and their linear combination (black curve). (**a**) Four curves with β=1.5 (blue dashed curve), β=1.1 (red dashed curve), β=0.1 (gray dashed curve) and their linear combination (black curve). (**b**) With $b_{1}=1\times 10^{-5}$, $b_{2}=1\times 10^{-3}$ and $b_{3}=1-b_{1}-b_{2}$, both with q=1.11 (case M=3).

Let us focus on two specific particular cases, namely M=2 with $q_{1}=q_{2}\equiv q$, and M=3 with $q_{1}=q_{2}=q_{3}\equiv q$; $\beta_{q_{1}}\equiv \beta_{1}$, $\beta_{q_{2}}\equiv \beta_{2}$, $\beta_{q_{3}}\equiv \beta_{3}$, and $Z_{q_{k}}\equiv Z_{k}$. It follows that

(22) $$p\left(x\right)=b_{1}\frac{e_{q}^{-\beta_{1}\;x}}{Z_{1}}+b_{2}\frac{e_{q}^{-\beta_{2}\;x}}{Z_{2}}$$

with $b_{2}=1-b_{1}$, $1/Z_{1}=\beta_{1}\left(2-q\right)$, and $1/Z_{2}=\beta_{2}\left(2-q\right)$, and

(23) $$p\left(x\right)=b_{1}\frac{e_{q}^{-\beta_{1}\;x}}{Z_{1}}+b_{2}\frac{e_{q}^{-\beta_{2}\;x}}{Z_{2}}+b_{3}\frac{e_{q}^{-\beta_{3}\;x}}{Z_{3}}$$

with $b_{3}=1-b_{1}-b_{2}$, $1/Z_{1}=\beta_{1}\left(2-q\right)$, $1/Z_{2}=\beta_{2}\left(2-q\right)$ and $1/Z_{3}=\beta_{3}\left(2-q\right)$. See Figure 3 and Figure 4.

**Figure 5:**
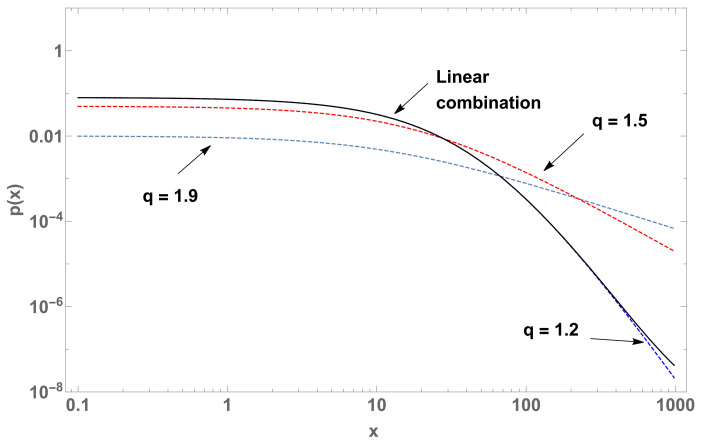
p(x) (log-log plot) of four curves (case M=3) with parameters β=0.1, q=1.2 (blue dashed curve), q=1.5 (red dashed curve), q=1.9 (gray dashed curve), and their linear combination (black curve) with $b_{1}=1\times 10^{-5}$, $b_{2}=1\times 10^{-3}$ and $b_{3}=1-b_{1}-b_{2}$.

In Figure 4 (M=3), we fix the value $q_{k}=1.11$ for k=1,2,3. Another illustration of the linear combination consists of fixing the value $\beta_{q_{k}}=\beta$ for k=1,2,3 and using three different values for $q_{k}$. In the case illustrated in Figure 5, the linear combination remains close to the curve corresponding to (q,β)=(1.2,0.1).

(24) $$p\left(x\right)=b_{1}\frac{e_{q_{1}}^{-\beta \;x}}{Z_{q_{1}}}+b_{2}\frac{e_{q_{2}}^{-\beta \;x}}{Z_{q_{2}}}+b_{3}\frac{e_{q_{3}}^{-\beta \;x}}{Z_{q_{3}}}$$

with $b_{3}=1-b_{1}-b_{2}$, $1/Z_{q_{1}}=\beta \left(2-q_{1}\right)$, $1/Z_{q_{2}}=\beta \left(2-q_{2}\right)$ and $1/Z_{q_{3}}=\beta \left(2-q_{3}\right)$.

Linear combinations of this kind (either of *q*-exponentials, or of *q*-Gaussians) have been fruitfully used in [4,13,24,25].

## 4. Linear Combination of *q*-Entropies

A linear combination of *q*-entropies can be written as follows:

(25) $$S\left(\left\{p_{i}\right\}\right)=\sum_{k=1}^{M}c_{k}\;S_{q_{k}}\left(\left\{p_{i}\right\}\right)\;\;\left(q_{1}<q_{2}<\cdots <q_{M}\right)\;\;\;\left(c_{k}\geq 0\right)\;.$$

This expression is generically not normalized. If we happen to prefer normalization for some specific reason, it is enough to divide Equation (25) by $\sum_{k=1}^{M}c_{k}$.

With the constraints $\sum_{i}p_{i}-1=0$ and $\sum_{i}p_{i}E_{i}-U=0$, where *U* is the internal energy of the system and $\left\{E_{i}\right\}$ are the energy eigenvalues, we define the functional $f(\alpha_{1},\alpha_{2},\left\{p_{i}\right\})$ as follows:

(26) $$f\left(\alpha_{1},\alpha_{2},\left\{p_{i}\right\}\right)\equiv \sum_{k=1}^{M}c_{k}\;S_{q_{k}}\left(\left\{p_{i}\right\}\right)+\alpha_{1}\left(1-\sum_{i}p_{i}\right)+\alpha_{2}\left(U-\sum_{i}p_{i}E_{i}\right)\;.$$

Then, through extremization, we obtain

(27) $$\frac{\partial }{\partial p_{j}}f=0=\sum_{k}c_{k}\left\{\ln_{q_{k}}\frac{1}{p_{j}}-{\left(\frac{1}{p_{j}}\right)}^{1-q_{k}}\right\}-\alpha_{1}-\alpha_{2}E_{j}$$

hence

(28) $$E\left(p_{j}\right)=-\frac{\alpha_{1}}{\alpha_{2}}+\frac{1}{\alpha_{2}}\sum_{k}c_{k}\left\{\ln_{q_{k}}\frac{1}{p_{j}}-{\left(\frac{1}{p_{j}}\right)}^{1-q_{k}}\right\}.$$

We introduce convenient new variables, namely

(29) $$\alpha_{1}\equiv -\alpha_{2}\mu ,\;\;\alpha_{2}\equiv \beta \;.$$

This enables us to express $X_{j}\equiv \beta \left(E_{j}-\mu \right)$ as an explicit function of $p_{j}$, namely

(30) $$X_{j}=\sum_{k}c_{k}\left\{\ln_{q_{k}}\frac{1}{p_{j}}-{\left(\frac{1}{p_{j}}\right)}^{1-q_{k}}\right\}.$$

**Figure 6:**
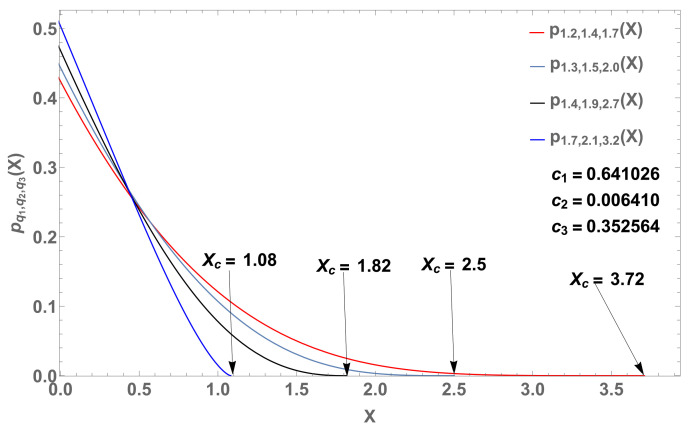
Four probability distributions $p_{q_{1},q_{2},q_{3}}\left(X\right)$ (*M* = 3) based on Equation (30) with $\left(c_{1},c_{2},c_{3}\right)=\left(0.641026,0.006410,0.352564\right)$. From (31), we respectively obtain the cutoff values $X_{c}=1.08$ for $\left(q_{1},q_{2},q_{3}\right)=\left(1.7,2.1,3.2\right)$ (blue curve), 1.82 for $\left(q_{1},q_{2},q_{3}\right)=\left(1.4,1.9,2.7\right)$ (black curve), 2.50 for $\left(q_{1},q_{2},q_{3}\right)=\left(1.3,1.5,2.0\right)$ (gray curve) and $X_{c}=3.72$ for $\left(q_{1},q_{2},q_{3}\right)=\left(1.2,1.4,1.7\right)$.

The cutoff condition, whenever present, is given by $\lim_{p_{j}\to 0}X\left(p_{j},q_{1},q_{2},\cdots ,q_{M}\right)\equiv X_{c}\left(q_{1},q_{2},\cdots ,q_{M}\right)$. For instance, for M=3, we have (see Figure 6)

(31) $$X_{c}\left(q_{1},q_{2},q_{3}\right)=\frac{c_{1}}{q_{1}-1}+\frac{c_{2}}{q_{2}-1}+\frac{c_{3}}{q_{3}-1},\;\;\left(1<q_{1}\leq q_{2}\leq q_{3}\right).$$

**Figure 7:**
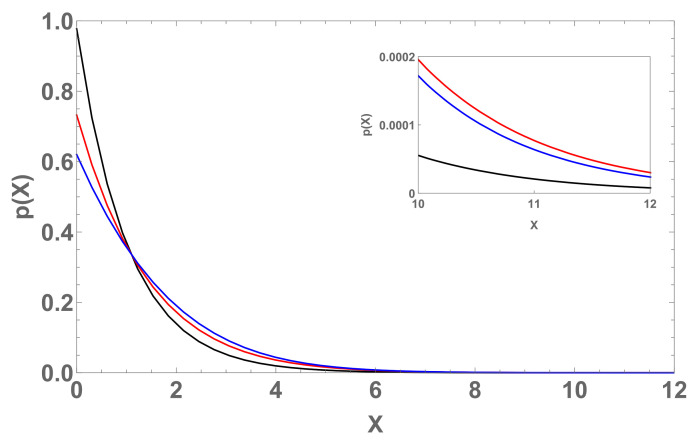
Three probability distributions p(X) based on Equation (33) with $c_{1}=0.3$ and q=1.01 hence $A_{q}=0.0238786$ (black curve), q=1.2 hence $A_{q}=0.6798077$ (red curve), and q=1.5 hence $A_{q}=2.3025270$ (blue curve).

The M=2 particular case of (25) has been focused on in [24]:

(32) $$S\left(\left\{p_{i}\right\}\right)=c_{1}S_{BG}\left(\left\{p_{i}\right\}\right)+c_{2}S_{q}\left(\left\{p_{i}\right\}\right)$$

where one of the entropies is the BG entropy (i.e., $q_{1}=1$), and the other one $S_{q}\left(\left\{p_{i}\right\}\right)$ corresponds to $q_{2}\equiv q\neq 1$. Then, we have (see Figure 7)

(33) $$p_{j}={\left\{aW\left(A_{q}e^{-\left(q-1\right)X_{j}}\right)\right\}}^{\frac{1}{q-1}}$$

where W(z) is the Lambert function, implicitly defined by $We^{W}=z$ (see, for instance, [26]), $A_{q}\equiv \frac{1}{a}e^{-\left(q-1\right)\left(1-\frac{c_{2}}{c_{1}\left(q-1\right)}\right)}$, $\alpha_{1}\equiv -\mu \alpha_{2}$, $\beta \equiv \frac{\alpha_{2}}{c_{1}}$ and $X_{j}\equiv \beta \left(E_{j}-\mu \right)$ (this definition of β differs from that in Equation (29)), with $a\equiv \frac{c_{1}}{c_{2}q}=\frac{c_{1}}{(1-c_{1})q}$. $A_{q}$ is determined via the normalization of the probabilities $\left\{p_{j}\right\}$, i.e.,

(34) $$\sum_{j}p_{j}=\sum_{j}{\left\{aW\left(A_{q}e^{-\left(q-1\right)X_{j}}\right)\right\}}^{\frac{1}{q-1}}=1.$$

In other words, $A_{q}$ implicitly depends on $\left(q,c_{1}\right)$. Whenever appropriate, we may go to the continuum limit. If it is allowed to consider X≥0, we have

(35) $$\int_{0}^{\infty }{\left\{aW\left(A_{q}e^{-(q-1)X}\right)\right\}}^{\frac{1}{q-1}}dX=1,$$

hence

(36) $$qa^{-\frac{1}{q-1}}=W{\left(A_{q}\right)}^{\frac{1}{q-1}}\left[q+W(A_{q})\right].$$

This expression determines *a* as an explicit function of $\left(q,A_{q}\right)$.

It is known that, in nonextensive statistical mechanics [27], the constraints under which the entropy is optimized might be chosen with escort distributions, namely, $\sum_{i}p_{i}-1=0$ and $\frac{\sum_{i}p_{i}^{q}E_{i}}{\sum_{i}p_{i}^{q}}-U_{q}=0$. We then have

(37) $$\tilde{f}\left(\alpha_{1},\alpha_{2},\left\{p_{i}\right\}\right)\equiv c_{1}S_{BG}\left(\left\{p_{i}\right\}\right)+c_{2}S_{q}\left(\left\{p_{i}\right\}\right)+\alpha_{1}\left(1-\sum_{i}p_{i}\right)+\alpha_{2}\left[U_{q}-\frac{\sum_{i}p_{i}^{q}E_{i}}{\sum_{i}p_{i}^{q}}\right]$$

hence

(38) $$p_{j}={\left\{ae_{q}^{\left(q-1\right)}W\left(B_{q}\;e_{q}^{-\left(q-1\right)X_{j}}\right)\right\}}^{\frac{1}{q-1}}\;,$$

where $X_{j}\equiv \beta^{\prime }\left(E_{j}-\mu \right)$ with $\beta^{\prime }$ defined as

(39) $$\beta^{\prime }\equiv \frac{\beta }{\sum_{j}p_{j}^{q}+\left(1-q\right)\beta U_{q}}$$

with $\beta \equiv \frac{\alpha_{2}}{c_{1}}$. Clearly, $B_{q}$ is determined by

(40) $$\sum_{j}p_{j}=\sum_{k}{\left\{a\;e_{q}^{\left(q-1\right)}W\left(B_{q}e_{q}^{-\left(q-1\right)X_{j}}\right)\right\}}^{\frac{1}{q-1}}=1\;.$$

Let us remind at this point that extremizing $S_{q}$ with standard constraints is equivalent to extremizing $S_{2-q}$ with escort constraints. The equivalence implies in doing the transformation q→2−q [27,28].

Let us address now the concavity/convexity of $S\left\{p_{i}\right\}$. We illustrate with the linear combination of two (M=2) *q*-entropies with $q_{1}$ and $q_{2}$, assuming $p_{1}\equiv p_{2}\equiv \cdots \equiv p_{\left(W-1\right)}\equiv p$ and $p_{W}=1-\left(W-1\right)p$. In other words, we consider

(41) $$\begin{array}{c} \begin{array}{cc} S_{q_{1},q_{2}}\left(p\right)=\;\;\; & c_{1}\left[\left(W-1\right)p\ln_{q_{1}}\left(\frac{1}{p}\right)+\left(1-\left(W-1\right)p\right)\ln_{q_{1}}\left(\frac{1}{1-(W-1)p}\right)\right]+\\ \; & c_{2}\left[\left(W-1\right)p\ln_{q_{2}}\left(\frac{1}{p}\right)+\left(1-\left(W-1\right)p\right)\ln_{q_{2}}\left(\frac{1}{1-(W-1)p}\right)\right]. \end{array} \end{array}$$

The study of concavity of (41) can be done in the $\left(q_{1},q_{2}\right)$ space, taking also into consideration the regions of non admissibility in which the entropy is neither concave nor convex.

**Figure 8:**
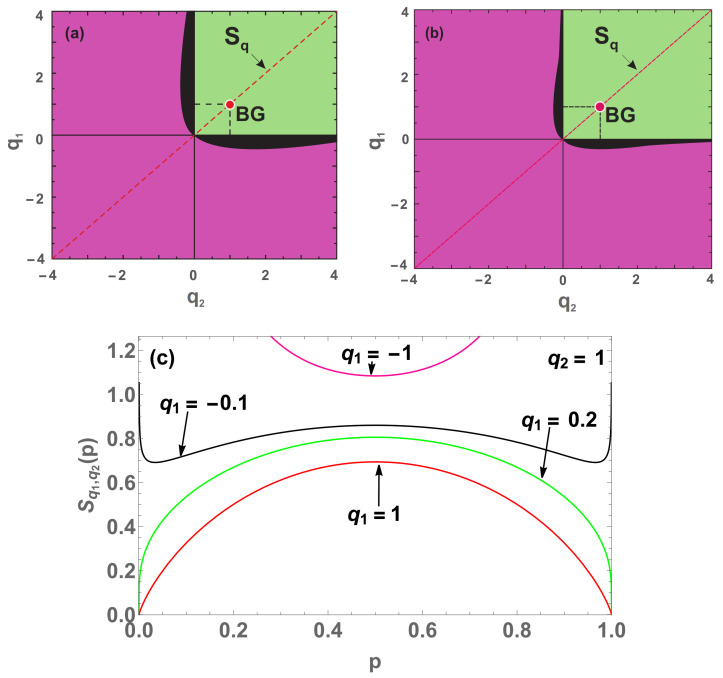
Concavity/convexity mapping for (41) with $\left(c_{1},c_{2}\right)=\left(0.48,0.52\right)$, W=2 (**a**) and W=3 (**b**). The green (pink) region represents all points whose entropy (41) is concave (convex). The black region represents all points whose entropy is neither concave nor convex, having two local minima points and a local maximum in between (a global maximum point at p=0.5 and divergences at p=0 and p=1). On the red point is localized the Boltzmann–Gibbs entropy and over the red dashed line cutting the origin, we have all the $S_{q}$ entropies. On the concave (convex) region we have $S_{q},\;q>0$ (q<0). (**c**) Four (W=2) entropies with $q_{2}=1$, and $q_{1}=1$ (blue curve), $q_{1}=0.2$ (green curve), $q_{1}=-0.1$ (black curve) and $q_{1}=-1$ (pink curve).

We clearly note that when W=3 (see Figure 8b), the black region is reduced compared to the W=2 case (Figure 8a). This result tends to suggest that the black region tends to disappear at W→∞, while the pink (convex) region predominates.

## 5. Other Departures—Two-Indices Entropies

We focus here on other type of departures from pure *q*-exponentials, originated now from two-indices nonadditive entropies which recover $S_{q}$ as particular instances.

### 5.1. $S_{q,\delta }$

From [14], we have

(42) $$S_{q,\delta }\equiv \sum_{i=1}^{W}p_{i}{\left[\ln_{q}\frac{1}{p_{i}}\right]}^{\delta }\;\;\;\left(q\in R;\;\delta >0\right)\;.$$

We verify that $S_{q,1}=S_{q}$. Extremization of $S_{q,\delta }$ under usual constraints yields

(43) $$E\left(p_{j}\right)=-\frac{\alpha_{1}}{\alpha_{2}}+\frac{1}{\alpha_{2}}\left\{{\left[\ln_{q}\frac{1}{p_{j}}\right]}^{\delta }-\delta {\left(\frac{1}{p_{j}}\right)}^{1-q}{\left[\ln_{q}\frac{1}{p_{j}}\right]}^{\delta -1}\right\}\;.$$

Through (29), we have

(44) $$X_{j}=\left\{{\left[\ln_{q}\frac{1}{p_{j}}\right]}^{\delta }-\delta {\left(\frac{1}{p_{j}}\right)}^{1-q}{\left[\ln_{q}\frac{1}{p_{j}}\right]}^{\delta -1}\right\}\;.$$

**Figure 9:**
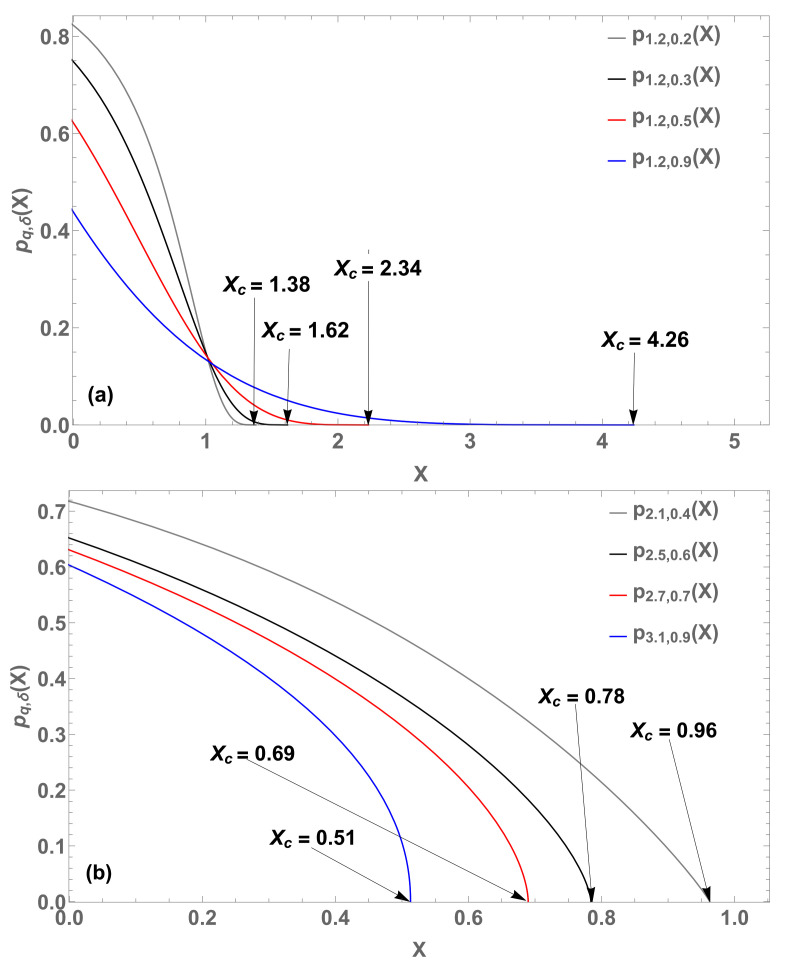
Illustrative probability distributions $p_{q,\delta }\left(X\right)$. (**a**) q=1.2 and δ=0.2 hence, through (45), $X_{c}=1.38$ (gray curve); δ=0.3, hence $X_{c}=1.62$ (black curve); δ=0.5 hence $X_{c}=2.34$ (red curve) and finally, δ=0.9 hence $X_{c}=4.26$ (blue curve); (**b**) (q,δ)=(3.1,0.9) hence $X_{c}=0.51$ (blue curve); (q,δ)=(2.7,0.7) hence $X_{c}=0.69$ (red curve); (q,δ)=(2.5,0.6) hence $X_{c}=0.78$ (black curve); and (q,δ)=(2.1,0.4) hence $X_{c}=0.96$ (gray curve).

Taking into account the transformation q→2−q mentioned below Equation (40), the cutoff occurs for q>1, and $X_{c}\left(q,\delta \right)$ is given by (see Figure 9)

(45) $$X_{c}\left(q,\delta \right)={\left(q-1\right)}^{-\delta }\;\;\left(q>1\right).$$

We verify that $p_{q,\delta }\left(X\right)$ is single-valued for q≥δ and multi-valued otherwise.

**Figure 10:**
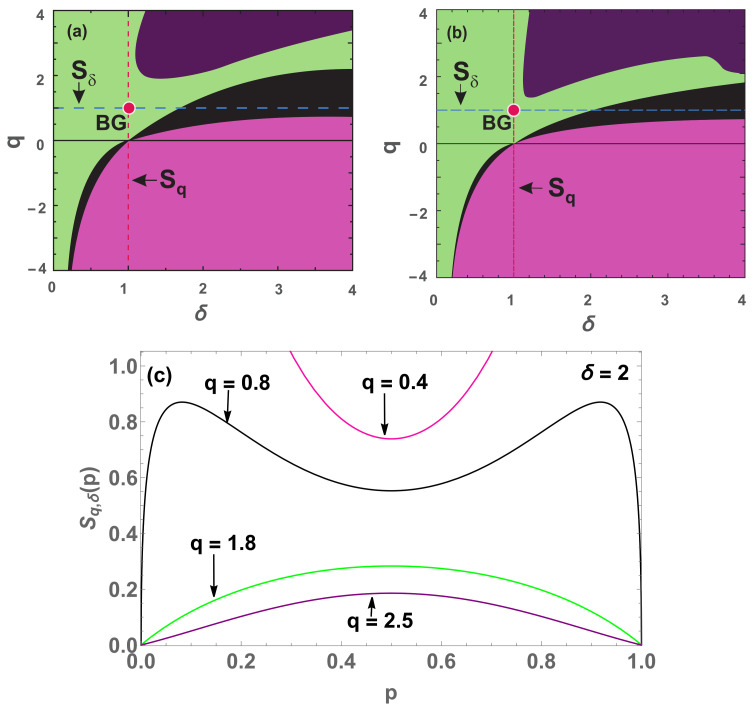
Concavity/convexity regions for $S_{q,\delta }$ (46) (**a**) W=2. (**b**) W=3. The green (pink) region represents all points whose entropy (41) is concave (convex). The black region represents all points whose entropy is neither concave nor convex, having two local maxima ( inflexion) points and another local minimum (maximum) in between. The points of transition at δ=2 are: $q_{c}=1/2$ (both W=2 and W=3) (pink↔black); $q_{c}=4/3$ (W=2) and $q_{c}∼0.98$ (W=3) (black↔green) and $q_{c}=2$ (both cases) (black↔purple). At q=1, we have the transition from non concave to concave at $\delta_{c}=1+\ln2$ (W=2) and for W=3, we have $\delta_{c}<1+\ln3$. The blue dashed horizontal line represents $S_{\delta }$, while the red dashed vertical line represents all $S_{q}$ entropies, and the red point is the BG entropy. (**c**) Four cases (W=2) for δ=2 with the respective colors: q=0.4 and q=1.8 (convex and concave regions respectively); q=0.8 (black region) and q=2.5 (purple region) (non concave and non convex regions).

**Figure 11:**
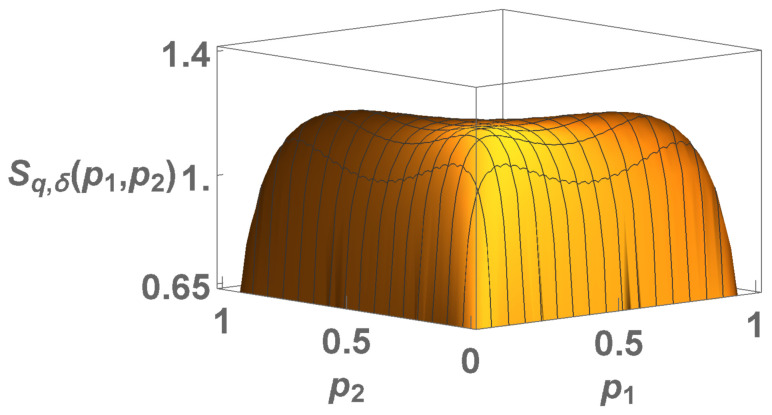
Plot for $S_{q,\delta }$ with W=3, q=1 and δ=1+ln3. We clearly observe that $\delta_{c}=1+\ln W$ is not valid here, because in this value, the entropy is not concave, much less the values close to this.

Let us now consider the case $p_{1}\equiv p_{2}\equiv \cdots \equiv p_{\left(W-1\right)}\equiv p$ and $p_{W}=1-\left(W-1\right)p$ hence

(46) $$S_{q,\delta }\left(p\right)=\left(W-1\right)p{\left[\ln_{q}\left(\frac{1}{p}\right)\right]}^{\delta }+\left(1-\left(W-1\right)p\right){\left[\ln_{q}\left(\frac{1}{1-(W-1)p}\right)\right]}^{\delta }\;,$$

where $p\in \left[0,\frac{1}{W-1}\right]$. This expression will help us to study the concavity/convexity of the entropy for increasing values of *W*. See Figure 10 and Figure 11.

The black region is clearly reduced for W=3 (see Figure 10b), but the purple region at, for example, δ=3.8 and q=2.15, invades the concave region. It is not excluded that the purple region gradually expands with *W* in such way that it approaches the black region.

We noticed that an inadvertence occurred in [14]. Indeed, it was therein indicated that, for all entropies $S_{\delta }$, it would be $\delta_{c}\left(W\right)=1+\ln W$, but this is not exactly so in some cases. As we verify in what follows, we always have $\delta_{c}\in \left(\ln W,1+\ln W\right]$. Therefore, the formula in [14] constitutes an upper bound of $\delta_{c}$.

The probability is limited by $p\geq \frac{1}{W-1}$. Numerically, we analyze the plot $1/\ln W\times \delta_{c}-\ln W$. If it was $\delta_{c}=1+\ln W$ for all entropies $S_{\delta }$, we should obtain $\delta_{c}-\ln W=1$ for all values of *W*, which is not the case.

The interpretation of $\delta_{c}$ is given by the transition green ↔ black; no transition black ↔ pink appears to exist.

**Figure 12:**
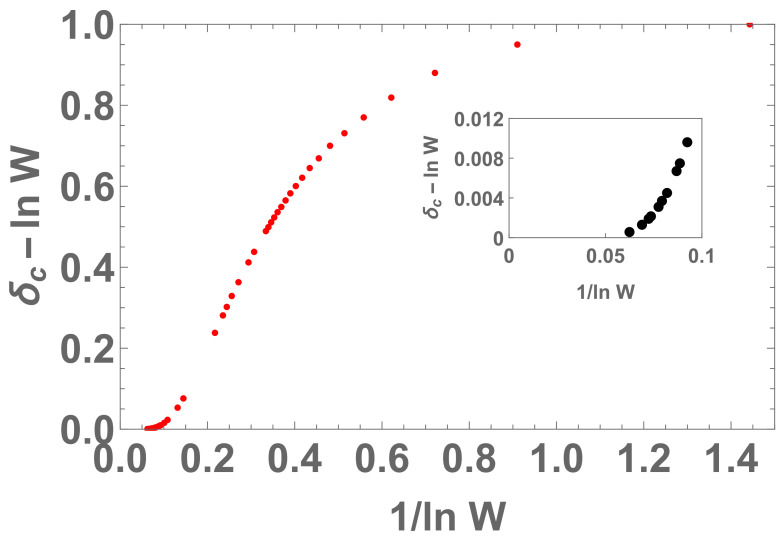
Plot for $1/\ln W\times \delta_{c}-\ln W$ with $W_{max}=9\times 10^{6}$. Here, $\delta_{c}\in \left(\ln W,1+\ln W\right]$. In the inset, we indicate the behavior of that function closer to origin.

**Figure 13:**
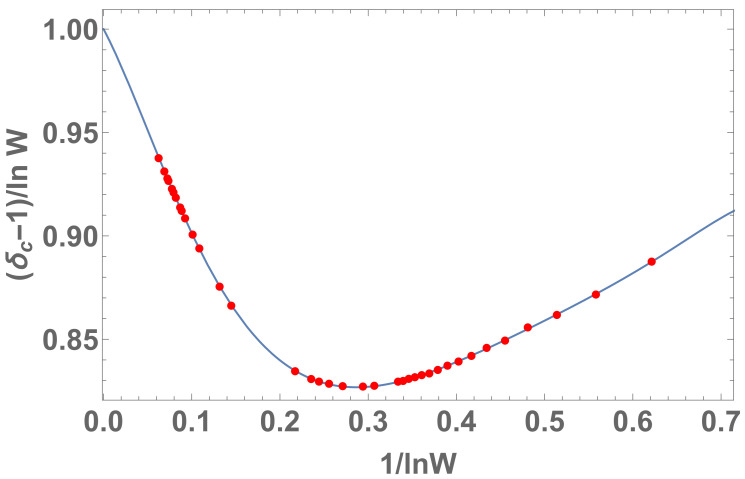
Plot for $1/\ln W\times (\delta_{c}-1)/\ln W$ with $W_{max}=9\times 10^{6}$. The regression by excluding the W=2 and W=3 points yields an 8th degree polynomial of x≡1/lnW, namely $f\left(x\right)\approx 1-0.794252x-6.20252x^{2}+60.9556x^{3}-223.39x^{4}+466.1x^{5}-588.297x^{6}+420.626x^{7}-130.677x^{8}$. It means that, when W→∞ we have x→0, thus $\lim_{x\to 0}f\left(x\right)=1$, therefore $\delta_{c}∼1+\ln W$ which diverges at infinity.

We notice in Figure 10, Figure 11, Figure 12 and Figure 13 that the divergence of $\delta_{c}$ in the limit W→∞ means that $S_{\delta }$ is concave in the thermodynamic limit for any positive δ.

### 5.2. Borges–Roditi Entropy $S_{q,q^{\prime }}^{BR}$

Borges and Roditi [15] extended the entropy $S_{q}$ as follows:

(47) $$S_{q,q^{\prime }}^{BR}=\frac{\sum_{i=1}^{W}p_{i}^{q}-\sum_{i=1}^{W}p_{i}^{q^{\prime }}}{q^{\prime }-q}\;,\;\;\left(\left(q,q^{\prime }\right)\in R^{2}\right)\;,$$

with $S_{q,1}^{BR}=S_{1,q}^{BR}=S_{q}$, where BR stands for Borges–Roditi; notice that $S_{q,q^{\prime }}^{BR}=S_{q^{\prime },q}^{BR}\;\;$.

Extremization with usual constraints, and using (29), we have:

(48) $$X_{j}=\frac{1}{q^{\prime }-q}\left(qp_{j}^{q-1}-q^{\prime }p_{j}^{q^{\prime }-1}\right)\;.$$

**Figure 14:**
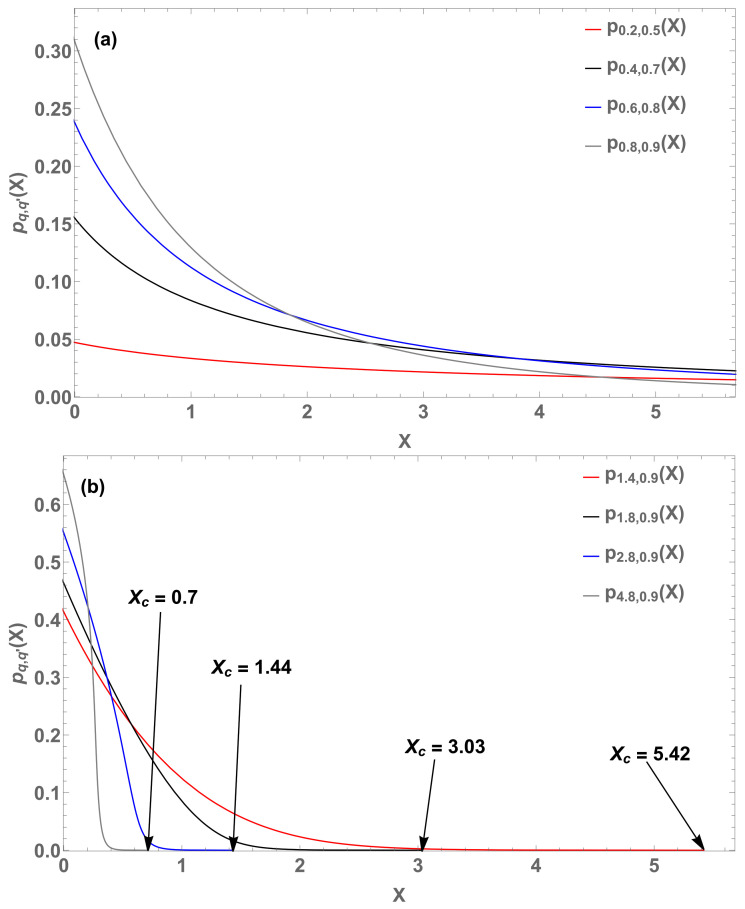
Eight illustrative Borges–Roditi probability distributions. (**a**) $\left(q,q^{\prime }\right)=\left(0.2,0.5\right)$ (red curve); $\left(q,q^{\prime }\right)=\left(0.4,0.7\right)$ (black curve); $\left(q,q^{\prime }\right)=\left(0.6,0.8\right)$ (blue curve), and $\left(q,q^{\prime }\right)=\left(0.8,0.9\right)$ (gray curve). (**b**) $\left(q,q^{\prime },X_{c}\right)=\left(1.4,0.9,5.42\right)$ (red curve), $\left(q,q^{\prime },X_{c}\right)=\left(1.8,0.9,3.03\right)$ (black curve), $\left(q,q^{\prime },X_{c}\right)=\left(2.8,0.9,1.44\right)$ (blue curve), and $\left(q,q^{\prime },X_{c}\right)=\left(4.8,0.9,0.7\right)$ (gray curve).

For $q,q^{\prime }<1$, *p* monotonically decreases to zero when *X* increases to infinity. For $q,q^{\prime }>1$, *p* is multivalued, hence physically inadmissible. For $q<1,\;q^{\prime }>1$ (hence, for $q>1,q^{\prime }<1$ ), *p* is single-valued and exhibits a cutoff at $X_{c}$. See Figure 14 for typical examples.

Let us focus now on the concavity of $S_{q,q^{\prime }}^{BR}$. By considering the same case that led to Equation (46), we obtain here

(49) $$S_{q,q^{\prime }}\left(p\right)=\frac{1}{q^{\prime }-q}\left[\left(W-1\right)p^{q}+{\left(1-\left(W-1\right)p\right)}^{q}-\left(W-1\right)p^{q^{\prime }}-{\left(1-\left(W-1\right)p\right)}^{q^{\prime }}\right]\;.$$

**Figure 15:**
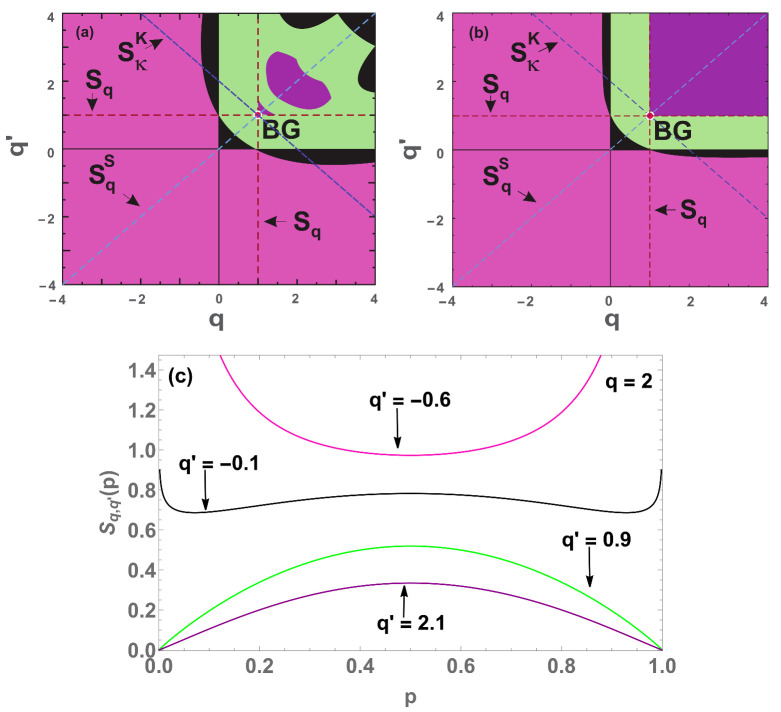
Concavity/convexity for $S_{q,q^{\prime }}^{BR}$ (49) with (**a**) W=2 and (**b**) W=3. The green (pink) region represents all points whose entropy (49) is concave (convex). The black (purple) region represents all points whose entropy is neither concave nor convex, having two local maxima (inflexion) points and another local minimum (maximum) in between. The red dashed vertical lines represent all $S_{q}$ entropies and the red point is the BG entropy, while the light (dark) blue lines represents all Shafee $S_{q}^{S}$ (Kaniadakis $S_{\kappa }^{K}$) entropies [29,30]. (**c**) Four illustrative cases (W=2) with q=2 and its respective colors: $q^{\prime }=-0.6$ and $q^{\prime }=0.9$ (pink and green regions respectively ); q=−0.1 (black region) and q=2.1 (purple region).

The purple region undergoes a slight change whether we compare the Figure 15a (W=2) and Figure 15b (W=3), although it appears that the rectangular purple region at W=3 does not increase for W>3. Indeed, if it did that, it would affect the BG and $S_{q}$ entropies whose convexity/concavity are known. With respect to the black region, the fact of that region shrinks from W=2 to W=3 suggests that it possibly disappears in W→∞.

### 5.3. $S_{q,q^{\prime }}$

On the basis of some algebraic properties, $S_{q}$ has been generalized in [16,31,32]:

(50) $$S_{q,q^{\prime }}=\sum_{i=1}^{W}p_{i}\ln_{q,q^{\prime }}\frac{1}{p_{i}}$$

with

(51) $$\ln_{q,q^{\prime }}z\equiv \frac{1}{1-q^{\prime }}\left[\exp\left(\frac{1-q^{\prime }}{1-q}\left(z^{1-q}-1\right)\right)-1\right]\;.$$

We verify that $\ln_{q,1}=\ln_{1,q}=\ln_{q}$, hence $S_{q,1}=S_{1,q}=S_{q}$. with $S_{q,1}=S_{1,q}=S_{q}$. Clearly, we can reformulate (51) in terms of $\ln_{q}$ such that

(52) $$\ln_{q,q^{\prime }}z=\frac{1}{1-q^{\prime }}\left[\exp\left(\left(1-q^{\prime }\right)\ln_{q}z\right)-1\right]\;.$$

The reformulated version of the extremized entropy $S_{q,q^{\prime }}$ is written as

(53) $$X_{j}=\exp\left(\left(1-q^{\prime }\right)\ln_{q}\frac{1}{p_{j}}\right)\left[\frac{1}{1-q^{\prime }}-{\left(\frac{1}{p_{j}}\right)}^{1-q}\right]-\frac{1}{1-q^{\prime }}$$

The cutoff equation $X_{c}\left(q,q^{\prime }\right)$ is given by

(54) $$X_{c}\left(q,q^{\prime }\right)=\frac{1}{1-q^{\prime }}\left[e^{-\frac{1-q^{\prime }}{1-q}}-1\right],\;q>1.$$

**Figure 16:**
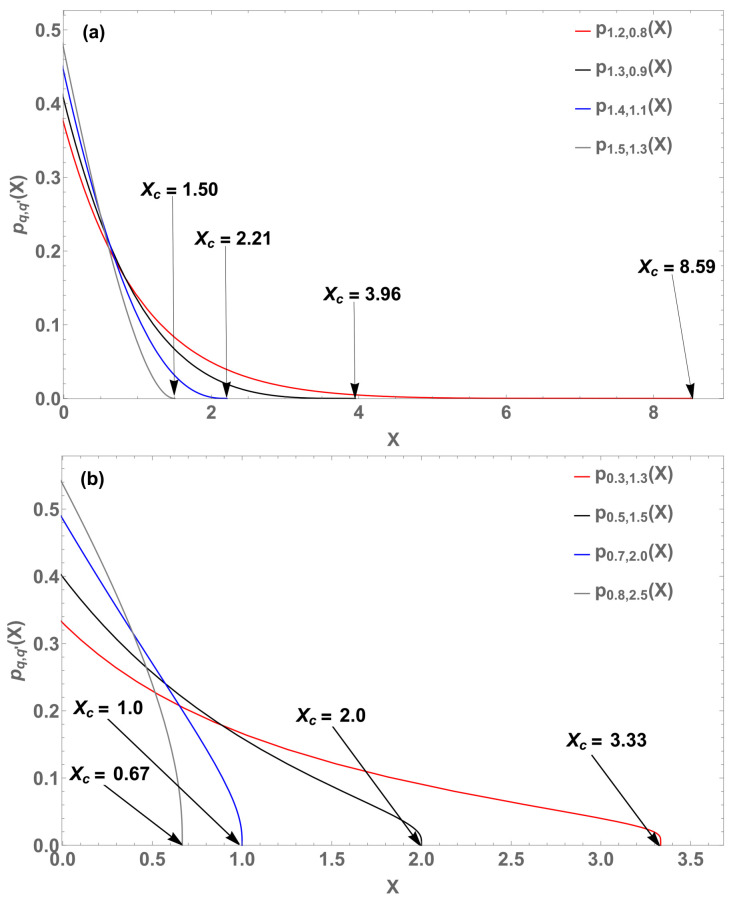
Eight illustrative probability distributions $p_{q,q^{\prime }}\left(X\right)$. (**a**) $\left(q,q^{\prime },X_{c}\right)=\left(1.5,1.3,1.5\right)$ (gray curve), $\left(q,q^{\prime },X_{c}\right)=\left(1.4,1.1,2.21\right)$ (blue curve), $\left(q,q^{\prime },X_{c}\right)=\left(1.3,0.9,3.96\right)$ (black curve), and $\left(q,q^{\prime },X_{c}\right)=\left(1.2,0.8,8.59\right)$ (red curve). (**b**) $\left(q,q^{\prime },X_{c}\right)=\left(0.8,2.5,0.67\right)$ (gray curve), $\left(q,q^{\prime },X_{c}\right)=\left(0.7,2.0,1.0\right)$ (blue curve), $\left(q,q^{\prime },X_{c}\right)=\left(0.5,1.5,2.06\right)$ (black curve), and $\left(q,q^{\prime },X_{c}\right)=\left(0.3,1.3,3.33\right)$ (red curve).

For q>1 and $0<q^{\prime }<1$, *p* is single-valued and exhibits a cutoff at $X_{c}$ (see Figure 16). For $q,q^{\prime }<1$, *p* is multi-valued, hence, it is inadequate for physical purposes. For 0<q<1 and $q^{\prime }>1$, *p* exhibits clearly a cutoff.

Analogously to (46), we write the Equation (50) as

(55) $$S_{q,q^{\prime }}\left(p\right)=\left(W-1\right)p\ln_{q,q^{\prime }}\left(\frac{1}{p}\right)+\left(1-\left(W-1\right)p\right)\ln_{q,q^{\prime }}\left(\frac{1}{1-(W-1)p}\right)\;.$$

**Figure 17:**
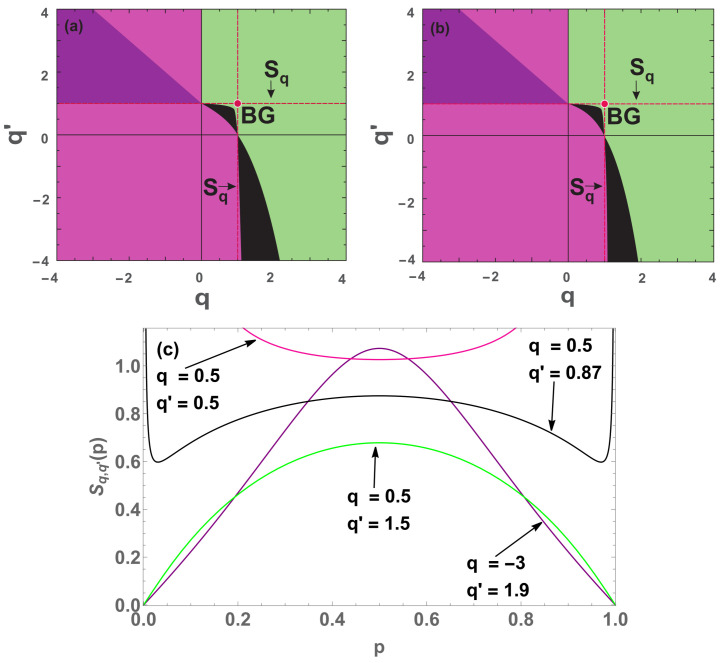
Concavity/convexity for $S_{q,q^{\prime }}$ (55) with (**a**) W=2 and (**b**) W=3. The green (pink) region represents all points whose entropy (55) is concave (convex). The black (purple) region represents all points whose entropy is neither concave nor convex, having two local maxima (inflexion) points and another local minimum (maximum) in between. The red dashed vertical line represents all $S_{q}$ entropies and the red point is the BG entropy. (**c**) Four cases (W=2) with the respective colors: with q=0.5, $q^{\prime }=0.5$ and $q^{\prime }=1.5$ (pink and green regions) and $q^{\prime }=0.87$ (black region), and $\left(q^{\prime },q\right)=\left(1.9,-3\right)$ (purple region).

In Figure 17a,b, we observe that the purple region appears to remain the same for all W≥2. In contrast, the black region for W=3 is slightly smaller than that for W=2, which suggests that, in W→∞, such a region might disappear. We checked for large values of *W*, and this scenario is confirmed. This happens in two different ways: the black region close to the BG point gradually disappears, being replaced by the pink (convex) region, and the black region in the negative part of $q^{\prime }$ also disappears, being replaced by the green (concave) region.

## 6. Conclusions

In summary, we have explored here various mathematical properties related to extensions of *q*-exponentials and *q*-entropies, including some double-index nonadditive entropies.

In the case of crossover statistics (Equation (7)), there are multiple changes in the slopes of the corresponding log-log plots. The values of the abscissa at which the relevant quantities make crossovers between two successive regimes are characterized by $x_{c}$, analytically calculated in all cases, as illustrated in Figure 1 and Figure 2.

When we consider linear combinations of normalized *q*-exponentials, we may focus on the influence of the $q_{k}$’s and of the $\beta_{k}$’s in Equation (18). For a single value of $\beta_{k}$ and various values for the $q_{k}$’s, the result might be close to one of the *q*-exponentials, whereas if we adopt a single value of $q_{k}$ and various values for the $\beta_{k}$’s, the outcome might be sensibly different from all the *q*-exponentials, as illustrated in Figure 3, Figure 4 and Figure 5.

With respect to the linear combination of *q*-entropies, it is generically impossible to have the probability distribution $p_{j}$ in Equation (30) as an explicit function of $X_{j}$. Notice, however, that we do have $X_{j}$ as an explicit function of $p_{j}$. This is in contrast with the case where we have linear combinations of the normalized *q*-exponentials. The final results for these two types of linear combinations clearly differ, as first shown in [24]. Let us emphasize that, consistently, the operations of linearly combining and entropic extremization do not commute.

In addition to that, for the linear combination of two nonadditive entropies (case *M* = 2), as well as for the three double-index nonadditive entropies (namely, $S_{q,\delta }$, $S_{q,q^{\prime }}^{BR}$ and $S_{q,q^{\prime }}$), we have studied their convexity/concavity in the indices-space. The results depend naturally on the total number of states (*W*). The limit W→∞ is particularly interesting, since it corresponds to the thermodynamical limit. We verify that, in the case of a linear combination of two *q*-entropies (M=2), the concave region remains one and the same for all values of *W*. Indeed, the value of *W* only affects the size of the convex region, as illustrated in Figure 8. It seems plausible that, in the W→∞ limit, the only possibilities which remain are either concave or convex. In what concerns $S_{q,\delta }$, $S_{q,q^{\prime }}^{BR}$ and $S_{q,q^{\prime }}$, regions in the indices-space exist, for a given value of *W*, where the entropy is concave, or convex, or none of them, as illustrated in Figure 10, Figure 15 and Figure 17. For all these three entropies, the region which is neither concave nor convex does not disappear even for W→∞. In particular, we have studied in detail the case of $S_{\delta }$ (q=1 and δ>0), and have obtained that convexity never emerges, ∀δ,∀W. A critical value $\delta_{c}\left(W\right)$ exists such that $S_{\delta }$ is concave for $\delta <\delta_{c}\left(W\right)$ and neither concave nor convex for $\delta >\delta_{c}\left(W\right)$; moreover, in the W→∞ limit, we verify that $\delta_{c}\left(W\right)∼\ln W$. The results displayed in the present paper could hopefully guide the use of entropies differing from $S_{q}$ for large classes of natural, artificial and social complex systems.
